# Dynamic regulation of HIF-1 signaling in the rhesus monkey heart after ischemic injury

**DOI:** 10.1186/s12872-022-02841-0

**Published:** 2022-09-11

**Authors:** Tao Wang, Ying Xiao, Jingyao Zhang, Fujia Jing, Guodan Zeng

**Affiliations:** Regenerative Medicine Research Center, West China Hospital, Sichuan University, Chengdu, 610041 Sichuan China

**Keywords:** HIF-1, Copper, Myocardial infarction, Angiogenesis, Glycolysis, ECM remodeling

## Abstract

**Background:**

Hypoxia inducible factor-1 (HIF-1) plays a key role in modulating post-infarct healing after myocardial ischemic injury through transcriptional regulation of hundreds of genes involved in diverse cardiac remodeling processes. However, the dynamic changes in HIF-1 target gene expression in the ischemic heart after myocardial infarction (MI) have not been well characterized.

**Methods:**

We employed a rhesus monkey model of MI induced by left anterior descending artery ligation and examined the expression pattern of HIF-1 target genes in the ischemic heart at 1, 7, and 28 days after injury by bulk RNA-sequencing analysis.

**Results:**

Myocardial transcriptomic analysis demonstrated a temporal-specific regulation of genes associated with the inflammatory response, cell proliferation, fibrosis and mitochondrial metabolism during the pathological progression of MI. HIF-1 target genes involved in processes related to glycolysis, angiogenesis, and extracellular matrix (ECM) remodeling also exhibited distinct expression patterns during MI progression. Copper concentrations were gradually decreased in the heart after ischemic injury, which was positively correlated with the expression of HIF-1-mediated angiogenic and glycolytic genes but negatively correlated with the expression of HIF-1-mediated ECM remodeling genes. Moreover, genes related to intracellular copper trafficking and storage were suppressed along with the loss of myocardial copper in the ischemic heart.

**Conclusions:**

This study demonstrated a dynamic, functional-specific regulation of HIF-1 target gene expression during the progression of MI. The fine-tuning of HIF-1 signaling in the ischemic heart may be relate to the alteration in myocardial copper homeostasis. These findings provide transcriptomic insights into the distinct roles of HIF-1 signaling in the heart after ischemic injury, which will help determine the beneficial cutoff point for HIF-1 targeted therapy in ischemic heart diseases.

**Supplementary Information:**

The online version contains supplementary material available at 10.1186/s12872-022-02841-0.

## Background

The pathophysiology of myocardial infarction (MI) has been greatly uncovered in the past few decades [1, 2]. Reduced blood supply caused by coronary artery occlusion leads to the massive loss of cardiomyocytes in the ischemic area of the heart and subsequently initiates a series of superbly orchestrated ventricular remodeling processes, such as inflammatory infiltration, metabolic reprogramming, angiogenesis and fibrosis [3–6]. Given that most of these remodeling processes in the ischemic heart are carried out in a microenvironment lacking oxygen and nutrients, hypoxia signaling plays a critical role in modulating postinfarction healing [7].

Hypoxia inducible factor-1 (HIF-1) is the major transcription factor that mediates hypoxia signaling in the ischemic heart [8, 9]. Under hypoxia or ischemia, the oxygen-sensitive α subunit (HIF-1α) is stabilized and translocates into the nucleus to form a transcriptional complex with the constitutively expressed β subunit (HIF-1β) and other cofactors, such as p300 and CREB-binding protein (CBP). The HIF-1 complex subsequently binds to core hypoxia response elements (HREs) to initiate transcription of its target genes [10]. Currently, hundreds of genes have been identified to be transcriptionally regulated by HIF-1, which are involved in a variety of biological processes, including apoptosis, angiogenesis, glucose metabolism and extracellular matrix (ECM) remodeling [11, 12]. Considering the multifunctional roles of HIF-1 target genes, fine-tuning of HIF-1 signaling in the ischemic heart is critical for the orderly progression of cardiac remodeling.

In fact, both experimental and clinical studies have shown that HIF-1α protein persistently accumulates in the ischemic heart during the progression of MI [13–17]; however, HIF-1 target genes are not continuously induced. For example, the expression of vascular endothelial growth factor A (*VEGFA*) in the ischemic heart was induced in the acute phase but not in the chronic phase of MI [13, 18–20]. Moreover, we previously employed a rhesus monkey model of MI by left anterior descending (LAD) artery ligation to examine the expression pattern of several HIF-1 target genes in the chronically infarcted heart. Intriguingly, the expression of insulin-like growth factor 2 (*IGF2*) and angiopoietin 2 (*ANGPT2*) were increased but *VEGFA* and fms-related tyrosine kinase 1 (*FLT1*, also known as *VEGF* receptor1) were decreased in the infarct area compared with the remote area or sham-operated control [17]. The differential expression of these genes at different phases of MI indicates selective regulation of HIF-1 transcriptional activity during the progression of MI; however, the underlying regulatory mechanism remains unclear.

Copper, an essential trace element in mammals, has been shown to be responsible for the selective regulation of HIF-1 transcriptional activity under hypoxic conditions [12, 17, 21–24]. In human umbilical vein endothelial cells (HUVECs) and primary cultures of neonatal rat cardiomyocytes, copper deprivation induced by a copper chelator, tetraethylenepentamine (TEPA), significantly blocks hypoxia-induced activation of a subset of HIF-1 target genes, such as the 19 kDa BCL2/adenovirus E1B protein-interacting protein 3 (*BNIP3*) and *VEGFA*, but has no effects on the expression of other HIF-1 target genes, such as *IGF2* [17, 22, 23]. Notably, recent studies have shown that copper concentrations in ischemic myocardium are gradually depleted with the progression of MI [17, 25], indicating that myocardial copper loss after long-term ischemic injury may be associated with the impaired HIF-1 transcriptional activity in the ischemic myocardium. Moreover, a recent study showed that blocking copper loss from the ischemic heart via cardiac-specific knockout of copper metabolism MURR1 domain1 (Commd1), a copper binding protein involved in copper efflux, resulted in significantly preserved expression of *Vegfa* and *Flt1* [26]*,* further indicating a potential role for copper in regulating HIF-1 target gene expression in the ischemic heart. Therefore, it is of great interest to investigate whether there is any correlation between myocardial copper levels and the expression of HIF-1 target genes after MI.

Although the multiple roles of HIF-1 in supporting the heart to cope with ischemic insult have been revealed in the past two decades, no systematic study on the expression pattern of HIF-1 target genes during the progression of MI has been undertaken, especially in primates. Here, we conducted a time-course study using RNA-sequencing (RNA-seq) to profile the dynamic expression of HIF-1 target genes in the ischemic heart at different times (1, 7, and 28 days) after LAD ligation in the rhesus monkey.

## Methods

### Experimental design

The objective of this study was to investigate the dynamic changes of HIF-1 signaling in the heart after ischemic injury. We used the rhesus monkey model of MI, which is an excellent surrogate for studying human myocardial disease. A total of 12 monkeys were randomly assigned to sham operated control group (n = 3) and MI group (n = 9). At 1, 7 and 28 days after the LAD ligation operation (n = 3 per each time point), the ischemic/infarct area of the heart was collected for the subsequent RNA-seq, RT-qPCR and immunofluorescent detection.

### Animals

Male rhesus monkeys (Macaca mulatta), aged 2–3 years old and weighing 4.5–6.0 kg, were obtained from Ping’an Animal Breeding Center in Chengdu, China. The animals were acclimatized to the laboratory conditions for at least one month in the Association for Assessment and Accreditation of Laboratory Animal Care accredited facility. Monkeys were housed in clean primate facilities and had access to food and water ad libitum, as well as seasonal fruit and vegetables. All monkeys were handled in strict accordance with good animal practice under the supervision of veterinarians and were monitored for evidence of disease and changes in attitude, appetite, or behavior suggestive of illness. All animal procedures were approved by the Institutional Animal Care and Use Committee at Sichuan University West China Hospital, following the guidelines of the US National Institutes of Health and the Animal Research Reporting In Vivo Experiments (ARRIVE).

### Myocardial infarction model and tissue preparation

As described in our previous studies [27, 28], the monkey’s heart was exposed via a left fourth intercostal thoracotomy incision (4–5 cm) in the chest wall. The LAD was occluded for 1 min followed by a 5 min reperfusion, and this occlusion-reperfusion cycle was repeated 3 times before the eventual permanent ligation. The sham-operated controls were subjected to the same surgical procedure with the exception of LAD artery occlusion and ligation.

Monkeys were sacrificed to obtain heart tissues at 1, 7, and 28 days after surgery. Each heart was examined carefully to separate the IA from the noninfarct area as distinguished by its pale appearance and stiff touch. The ischemic hearts were divided into several pieces, one for copper concentration determination, and the others were preserved in liquid nitrogen for RNA sequencing, RT-qPCR and immunofluorescence staining.

For induction of mouse model of MI, mice (8–10 weeks old, male) were subject to permanent ligation of the left anterior descending (LAD) coronary artery as described in our previous studies [25]. Mice were sacrificed at 1, 7, and 28 days after MI to obtain heart samples. The ischemic area (IA) and remote area (RA) of the heart were then separated for further RT-qPCR analysis.

### RNA-seq and data analysis

Total RNA was extracted from each sample using TRIzol reagent (Invitrogen, USA) according to the manufacturer’s instructions. The concentration and quality of each sample were measured by an Agilent Technologies 2200 TapeStation (Life Technologies, USA). Sequencing libraries of each RNA sample were prepared by using the NEBNext ultra directional RNA Library Prep kit, NEBNext Poly(A) mRNA magnetic isolation module and NEBNext multiplex oligos (NEB, USA) according to the protocol provided by the manufacturer and performed on a 2720 Thermal Cycler (Life Technologies, USA). Finally, the cDNA library was sequenced by a HiSeq X instrument (Illumina, USA). All quality control of sequencing data were evaluated and filtered by Fast-QC software. Salmon (version 1.3) was used for mapping reads to the rhesus monkey reference genome (Mmul_10, INSDC Assembly GCA_003339765.3) using the default settings with the GC Bias flag to correct for systematic biases. The Salmon Count matrix was then imported into DESeq2 for variance stabilizing transformation (VST) normalization and differential analysis of gene expression using the Wald T test for significance (|log2FC|> 1, FDR < 0.05). Gene Ontology (GO) functional enrichment analysis of differentially expressed genes (DEGs) was performed using the Database for Annotation, Visualization, and Integrated Discovery bioinformatics resources (DAVID v6.8).

For HIF-1 target gene analysis, we first intersected the collection of differentially expressed genes at different times after MI with the previously validated HIF-1 target gene set (Additional file  5: Table S3) [11], and identified a total of 89 HIF-1 target genes that were significantly changed (|log2FC|> 0.58 and *p* value < 0.05) during the progression of MI (Additional file  6: Table S4). GO functional analysis and gene set enrichment analysis (GSEA, v4.1.0) were then employed to examine the dynamic change in HIF-1-regulated biological processes after MI. The heatmap was generated using Multiexperiment Viewer (MeV, v4.9) and clustered by hierarchical k-means clustering based on the abundance profiles.

The expression patterns of HIF-1 target genes during the MI progression in mice were analyzed based on the GSE114695 and GSE151834 datasets obtained from the Gene Expression Omnibus (GEO) database, and visualized by heatmaps.

### Copper concentration determination

Copper concentrations were determined using a graphite furnace atomic absorption spectrophotometer (AAS, Thermo, USA). Fresh heart samples were washed with PBS containing 10 mmol L^−1^ EDTA (Sigma, USA) several times to eliminate the blood copper and subsequently digested with concentrated nitric acid (HNO_3_, Sigma, USA) at 50 °C overnight (< 80 mg wet tissue with 0.5 ml nitric acid). Copper concentrations were normalized to the wet weight of the heart (µg/g wet weight).

### Immunofluorescence staining

Frozen heart sections were fixed in 4% paraformaldehyde for 10 min, permeabilized with 0.1% Triton X-100 for 10 min at room temperature, and then blocked with 2% bovine serum albumin (BSA, Gentihold, USA) for 1 h at 37 °C. Then, the tissue slides were incubated overnight with the primary antibodies anti-Ki67 (MA5-14,520, Thermo Fisher, USA) and anti-collagen I (ab138492, Abcam, UK) at 4 °C and were subsequently incubated with the secondary antibody Alexa Fluor 568 goat anti-rabbit (A-11036, Thermo Fisher, USA) at 37 °C for 1 h. Replacement of the primary antibody with PBS served as a negative control. The nuclei were observed by DAPI counterstaining. Fluorescence images were collected by confocal microscopy (Nikon, Japan).

### Real-time quantitative polymerase chain reaction (RT–qPCR)

Total RNA was isolated using TRIzol (Invitrogen, USA), and 1 µg RNA was used for reverse transcription using a PrimeScript RT Reagent Kit (TaKaRa, JPN) according to the manufacturer’s instructions. RT–qPCR was performed by using TB Green Premix Ex Taq II (TaKaRa, JPN). The samples were processed using a Thermo Q6 Real-Time System. The gene expression level was analyzed relative to TBP using the 2^−Δ(ΔCt)^ method in each sample. Three replicates were run for each sample. The primer sequences are shown in Table 1.

**Table 1:** RT–qPCR primer sequences for monkeys and mice

Primer	Forward sequence (5’ to 3’)	Reverse sequence (5’ to 3’)
*Monkey*		
*CXCR1*	ATCCACAGATGGGGGATGATG	CAGGGCAAAGAGTAGGTCGG
*CDK1*	ATAATAAGCCGGGATCTGCC	CATGGCTACCACTTGACCTG
*COL1A1*	TGACGAGACCAAGAACTGCC	CAGGAGATTACCTCGACGCC
*COQ9*	CCCTCACAACATCCCGTCC	GGTGGCAGGTGCAGCTATGAT
*LDHA*	TCTGATTTCCGCCCACCTTT	TCACTTTGAGCCACTCCTGC
*ANGPT2*	CCACATCAAACTCAGCTAAGGAC	ATAATTGTCCACCCGCCTCC
*LOX*	CACAGGGTGCTGCTCAGATT	TGACAACTGTGCCATTCCCA
*P4HA1*	AATGACCCCTCGGAGACAGA	TACCGTCTCCAAGTCTCCTGT
*VEGF*	GAGCTTCCTACAGCACAACA	CCAGGACTTATACCGGGATTTC
*SLC2A1*	GCCTGAGTCTCCTCTACCCA	CAAGTGTCTGGACAGGGCAT
*TBP*	TGCTCACCCACCAACAGTTT	TGCTCTGACTTTAGCACCTGT
*Mouse*		
*Vegfa*	AGGCTGCTGTAACGATGAAG	TCTCCTATGTGCTGGCTTTG
*Slc2a1*	CAGTTCGGCTATAACACTGGTG	GCCCCCGACAGAGAAGATG
*Lox*	ACTTCCAGTACGGTCTCCC	AGTCTCTGACATCCGCCCTA
*Tbp*	CCTTGTACCCTTCACCAATGAC	ACAGCCAAGATTCACGGTAGA

### Statistical analysis

All data were analyzed by one-way ANOVA followed by Dunnett’s multiple comparison test (RNA-seq and qPCR) or Pearson correlation analysis (correlation analysis) by Prism (v9.0). The data from each experiment are expressed as the mean ± SD. *P* < 0.05 was considered significant.

## Results

### Temporal correlation between the myocardial transcriptome and pathophysiology in the ischemic heart after MI

**Fig. 1:**
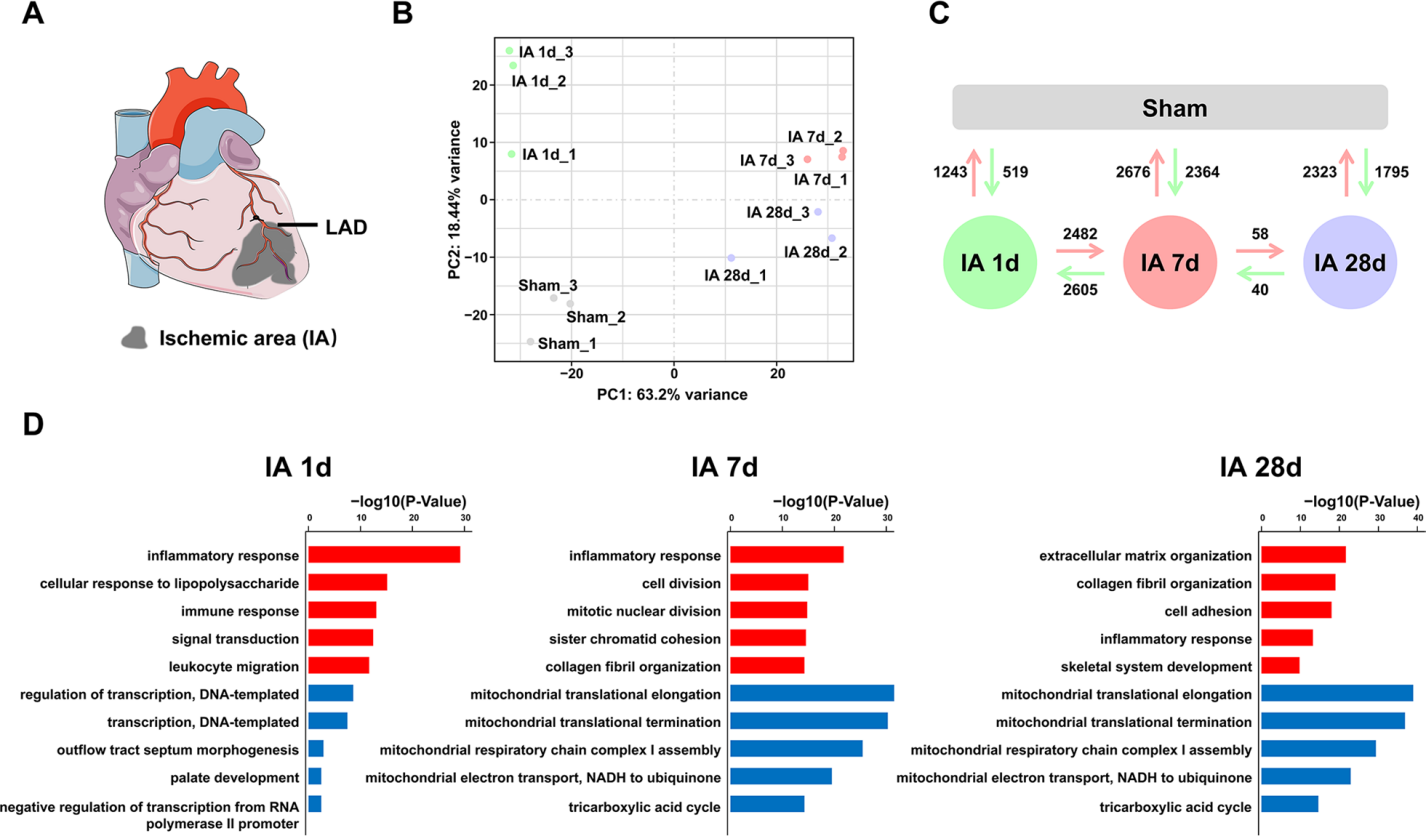
Transcriptomic changes in the ischemic heart during the progression of MI. **A** Schematic diagram of the ischemic area (IA) after LAD ligation in the rhesus monkey heart. **B** Principal component analysis of sham and IA samples at 1, 7, and 28 days after MI. **C** Comparison of the differentially expressed genes (DEGs, |log2FC|> 1 and FDR < 0.05) between the sham and different IA groups. **D** The list of the top five GO terms enriched by the upregulated (red) or downregulated (blue) DEGs in different IA groups compared with the sham group

We first characterize the transcriptomic changes in the ischemic heart at different phases of MI in rhesus monkeys. The ischemic area (IA) of the left ventricles at 1, 7, and 28 days after permanent LAD ligation or normal left ventricles from the sham-operated controls were collected for RNA-seq analysis (Fig. 1A). The principal component analysis (PCA) indicated a distinct transcriptomic signature of ischemic myocardium during the progression of MI (Fig. 1B). The number of differentially expressed genes (DEGs) between different groups is shown in Fig. 1C, and gene details are presented in Additional file  3: Table S1. Venn diagrams were used to show the number of common DEGs that were up- or down-regulated at different time points after MI (Additional file 1: Fig. S1A). Briefly, major transcriptional changes occurred within one week after ischemic injury by having the maximum DEGs in IA 7 d when comparing with sham-operated control or comparing with IA 1 d.

Gene Ontology (GO) functional analysis using DEGs between sham and each IA group unraveled their main biological functions, respectively (Fig. 1D). Specifically, inflammatory and immune responses were upregulated and the regulation of transcription was downregulated in IA 1 d. The activated processes in IA 7 d group were mainly involved in the inflammatory response, collagen fibril organization and processes associated with cell proliferation, and then shifted to ECM organization, collagen fibril organization, cell adhesion and skeletal system development in IA 28 d group. The downregulated genes in the IA 7 d group and IA 28 d group shared more than 80% similarity and were mainly enriched in processes related to mitochondrial metabolism.

**Fig. 2:**
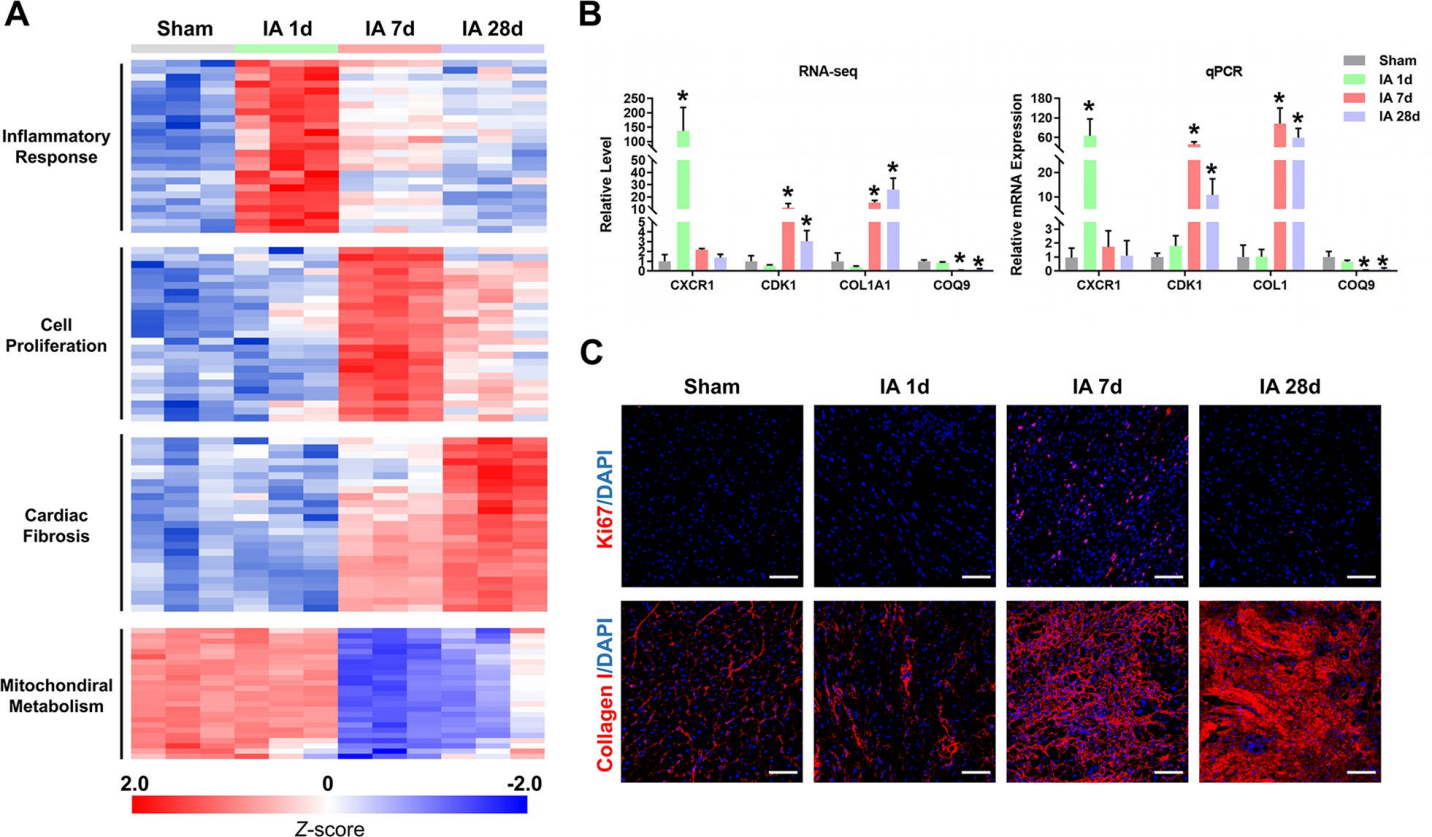
Relationship between the cardiac transcriptome and pathological changes after MI. **A** Heatmap of the top 25 significantly changed genes from each process, which exhibited obvious temporal specificity after MI. **B** The expression of mRNA levels determined by RNA-seq and qPCR. qPCR analysis was carried out to examine the expression pattern of representative genes from each process (*CXCR1*, *CDK1*, *COL1A1* and *COQ9*) on the same samples used for RNA-seq. n = 3, * *P* < 0.05, as determined by one-way ANOVA against the sham group. **C** Immunostaining of Ki67 (red) or Collagen I (red). Nuclei were counterstained with DAPI (blue). Scale bar, 100 μm

The expression pattern of the top 25 DEGs from each aforementioned processes exhibited obvious temporal specificity after ischemic injury (Fig. 2A, Additional file  4: Table S2). The expression of genes involved in inflammatory response, cell proliferation and cardiac fibrosis were sequentially activated in ischemic myocardium during MI progression. Additionally, genes related to mitochondrial metabolism were found to be continuously suppressed after 7 days of ischemic injury (Fig. 2A). RT-qPCR analysis of representative genes involved in each process, including C-X-C motif chemokine receptor 1 (*CXCR1*), cyclin-dependent kinase 1 (*CDK1*), collagen type I alpha 1 chain (*COL1A1*) and coenzyme Q9 (*COQ9*), further confirmed their expression patterns in RNA-seq (Fig. 2B). Immunostaining using anti-Ki67 antibody revealed a proliferative state in the ischemic heart at 7 days of MI, and immunostaining of type I collagen indicated the gradual formation of fibrotic scar in infarcted myocardium after 7 days of MI (Fig. 2C). Collectively, these observations demonstrate that there is a significant temporal-specific correlation between changes in the myocardial transcriptome and pathologies in the monkey hearts after MI, which is in good agreement with the current understanding of the pathophysiology of MI [2].

### Dynamic changes in the expression of HIF-1 target genes in the heart after ischemic injury

**Fig. 3:**
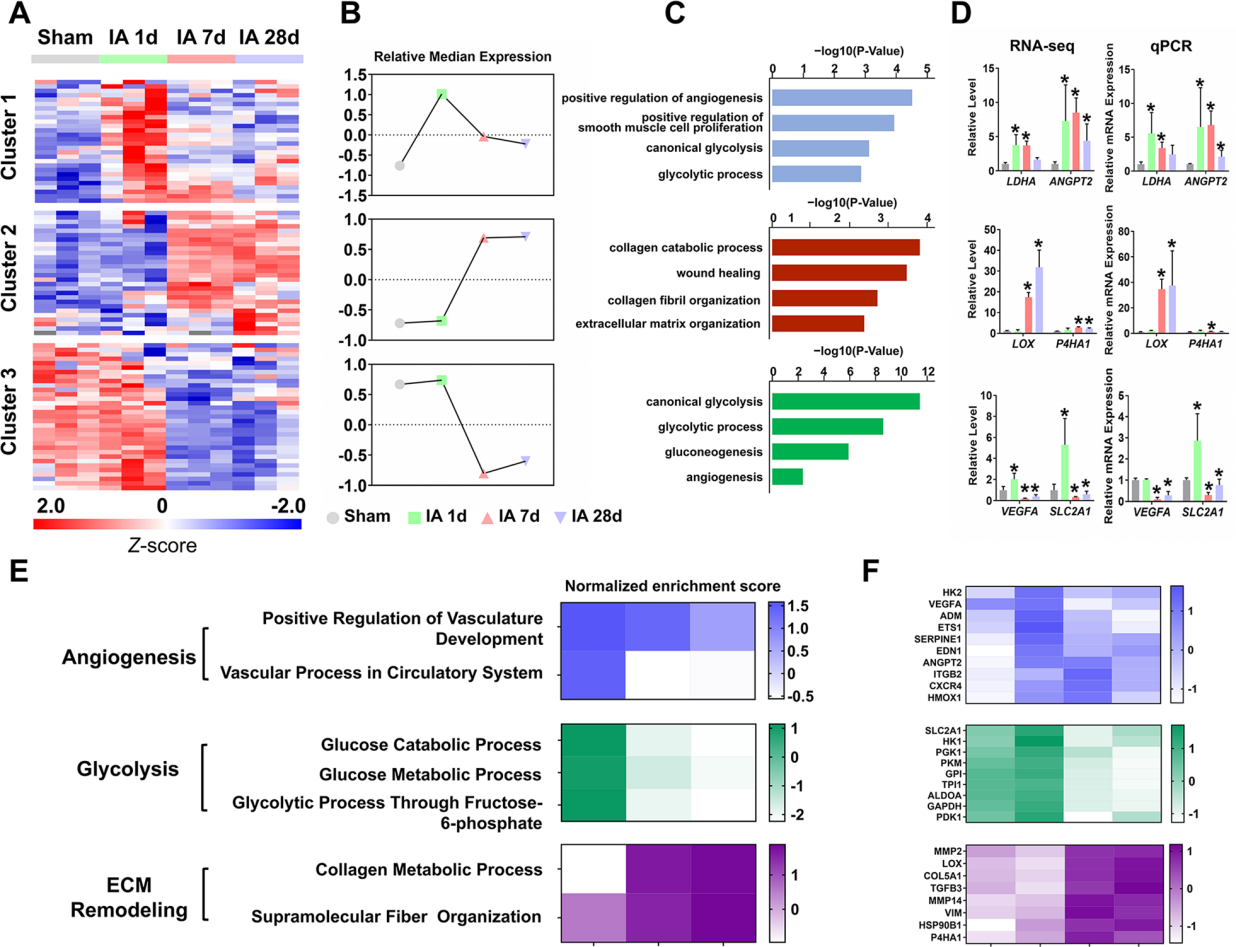
Dynamic changes in HIF-1 target genes in the ischemic heart after MI. **A** Heatmap of 89 significantly changed HIF-1 target genes after ischemic injury, which were segregated into 3 clusters based on their expression patterns using k-means clustering analysis. **B** Line chart of corresponding clusters. **C** Top 4 GO terms associated with each cluster. **D** The expression of mRNA levels determined by RNA-seq and qPCR. qPCR analysis was carried out to examine the expression pattern of two representative genes from each cluster (*LDHA* and *ANGPT2* from cluster 1, *LOX* and *P4HA1* from cluster 2, and *VEGFA* and *SLC2A1* from cluster 3) on the same samples used for RNA-seq. n = 3, * *p* < 0.05, as determined by one-way ANOVA against the sham group. **E** Heatmap of normalized enrichment scores (NES) of selected biological processes that were significantly enriched from GSEA. **F** Heatmap showing the dynamic changes in three subsets of HIF-1 target genes that were significantly enriched in glycolysis, angiogenesis and ECM remodeling

To understand the specific role of HIF-1 signaling in modulating postinfarction healing, we examined the transcriptional changes of HIF-1 target genes during the progression of MI based on a dataset of previously validated HIF-1 target genes (Additional file  5: Table S3) [11]. A total of 89 HIF-1 target genes were identified to be significantly changed (|log2FC|> 0.58 and *P* < 0.05) after ischemic injury (Additional file 6: Table S4). We then performed clustering analysis using *k*-means clustering and segregated HIF-1 target genes into 3 clusters based on their expression patterns (Fig. 3A, Table 2). In general, cluster 1 contained genes that were transiently upregulated at 1 day post-MI, while genes enriched in cluster 2 were upregulated at both 7 and 28 days after injury, and that in cluster 3 were downregulated (Fig. 3B). GO analysis was performed to identify related biological processes enriched by HIF-1 target genes in each cluster. Intriguingly, gene sets from clusters 1 and 3 were mainly associated with glycolysis and angiogenesis, while cluster 2 contained genes that were primarily enriched in processes related to ECM remodeling (Fig. 3C). qPCR analysis of two representative genes from each cluster (*LDHA* and *ANGPT2* from cluster 1, *LOX* and *P4HA1* from cluster 2, and *VEGFA* and *SLC2A1* from cluster 3) further confirmed their expression patterns (Fig. 3D). Furthermore, GO analysis of the differential expressed HIF-1 target genes at different times after MI showed that HIF-1-mediated angiogenic and glycolytic signaling in ischemic myocardium was significantly activated at 1 day but declined after 7 days of MI, whereas collagen metabolic processes were significantly activated after 7 days of MI (Additional file 1: Fig. S1B). Together, these results reveal dynamic, time-dependent regulation of HIF-1 target gene expression in the ischemic heart after MI.

**Table 2:** Significantly changed HIF-1 target genes in the ischemic heart

Cluster	Gene symbol						
1	*ABCB1*	*EDN1*	*HK2*	*KRT18*	*NAMPT*	*PFKFB3*	*STC2*
	*ADM*	*ENO1*	*HMOX1*	*LDHA*	*NDRG1*	*PLAUR*	*TFRC*
	*ANGPT2*	*ETS1*	*IGFBP2*	*MCL1*	*NPM1*	*PMAIP1*	*TGM2*
	*CDKN1A*	*FURIN*	*KDM3A*	*MET*	*PDGFA*	*SERPINE1*	*UGP2*
2	*BNIP3 L*	*COL5A1*	*CXCR4*	*IGF2*	*LOX*	*NT5E*	*SLC31A1*
	*CA9*	*CP*	*FN1*	*IGFBP3*	*LRP1*	*P4HA1*	*TGFA*
	*CCNG2*	*CTSD*	*HSP90B1*	*ITGB2*	*MMP14*	*P4HA2*	*TGFB3*
	*CD99*	*CXCL12*	*ID2*	*KDM4C*	*MMP2*	*PFKL*	*VIM*
3	*ABCG2*	*BNIP3*	*ENG*	*GPX3*	*MXI1*	*PDK1*	*SLC2A1*
	*ADRA1B*	*CITED2*	*FECH*	*GYS1*	*NOS2*	*PGK1*	*TPI1*
	*AK3*	*DDIT4*	*FLT1*	*HK1*	*NOS3*	*PKM*	*VEGFA*
	*ALDOA*	*EGLN1*	*GAPDH*	*IGFBP1*	*NR4A1*	*PPP5C*	
	*ANKRD37*	*EGLN3*	*GPI*	*KDM4B*	*P4HTM*	*RCOR2*	

Since HIF-1-controlled genes, particularly genes involved in glycolysis, angiogenesis and ECM remodeling, were differentially expressed in the heart following ischemic injury, HIF-1 signaling might play distinct roles at different phases of MI. Thus, we employed gene set enrichment analysis (GSEA) to investigate the dynamic changes in HIF-1-regulated biological processes in the ischemic heart (Fig. 3E). Consistently, glycolysis- and angiogenesis-related processes were significantly activated at 1 day post-MI but were gradually suppressed thereafter, whereas ECM remodeling processes were strongly induced at both 7 and 28 days after MI. Next, we extracted three subsets of HIF-1 target genes that were significantly enriched in these processes. As shown by heatmaps (Fig. 3F), 9 glycolysis-related genes (e.g., *SLC2A1*, *HK1*, *PGK1*, *PKM*, *GPI*, *TPI1*, *ALDOA*, *GAPDH* and *PDK1*), 10 angiogenesis-related genes (e.g., *HK2*, *VEGFA*, *ADM*, *ETS*, *SERPINE1*, *EDN1*, *ANGPT2*, *ITGB2*, *CXCR4* and *HMOX1*), and 8 ECM remodeling-related genes (e.g., *MMP2*, *LOX*, *COL5A1*, *TGFB3*, *MMP14*, *VIM*, *HSP90B1* and *P4HA1*) were identified to be specifically involved in mediating these distinct functions of HIF-1 signaling. Moreover, by analyzing two independent mouse RNA-seq datasets downloaded from the GEO database (GSE114695 and GSE151834) [29, 30], we found that the expression of the above-mentioned HIF-1 target genes also exhibited very similar patterns during the progression of MI in mice (Additional file  2: Fig. S2A). RT-qPCR analysis further confirmed the expression patterns of several representative HIF-1 target genes, including *Vegfa*, *Slc2a1* and *Lox*, in mouse model of MI (Additional file  2: Fig. S2B). Taken together, these findings indicate that HIF-1 signaling exerts diverse roles during the progression of MI, mainly by dynamically modulating processes related to glycolysis, angiogenesis and ECM remodeling.

### Myocardial copper homeostasis is associated with the differential expression of HIF-1 target genes

**Fig. 4:**
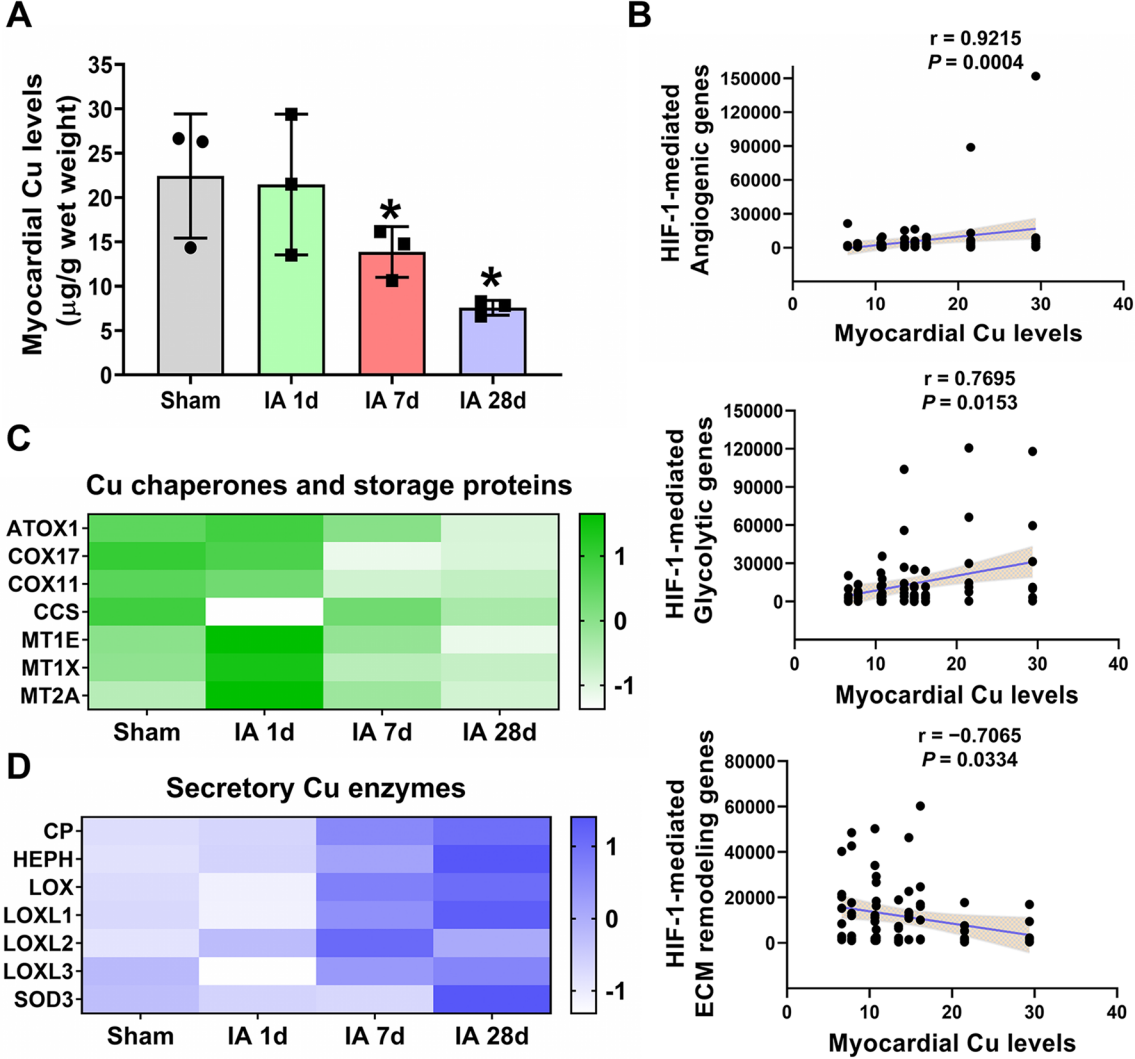
Changes in myocardial copper homeostasis. **A** Changes in myocardial copper concentrations at 1, 7, and 28 days after MI. **B** Pearson correlation analysis between myocardial copper levels and HIF-1-mediated glycolytic genes (left) or ECM remodeling genes (right). **C** Pearson correlation analysis between myocardial copper levels and HIF-1-mediated angiogenic genes (left) or *VEGFA* (right). **D** Dynamic changes in the expression of copper chaperones and storage proteins (left) and secretory copper enzymes (right)

To investigate the regulatory mechanism underlying the differential expression of HIF-1 target genes after MI, we determined the change in myocardial copper homeostasis since copper regulates the target gene selectivity of HIF-1 under hypoxic conditions [12, 17, 21–23]. Compared with the normal myocardium, copper concentrations in ischemic myocardium were depleted by 50% of the normal level at 7 days after injury and by 70% at 28 days (Fig. 4A), which was consistent with our previous findings in mice [25]. Correlation analysis demonstrated that copper levels in ischemic myocardium were positively correlated with the expression of HIF-1-mediated angiogenic genes (r = 0.9215, *P* = 0.0004) and glycolytic genes (r = 0.7695, *P* = 0.0153), but negatively correlated with the expression of HIF-1-mediated ECM remodeling genes (r = − 0.7065, *P* = 0.0334) (Fig. 4B). These data provide in vivo evidence that copper may selectively regulate the transcriptional activation of HIF-1 target genes.

To further understand the alterations in myocardial copper homeostasis in response to MI-induced myocardial copper loss, we first analyzed the dynamic change in genes involved in copper intracellular trafficking (Fig. 4C). Copper chaperones, including antioxidant 1 (*ATOX1*) and cytochrome *c* oxidase (CCO) assembly proteins (*COX17* and *COX11*), but not copper chaperone for superoxide dismutase (*CCS*), displayed a similar trend to changes in myocardial copper levels. In addition, the expression of metallothioneins (*MT1E*, *MT1X*, and *MT2A*), a family of cysteine-rich metal-binding proteins involved in intracellular copper storage and trafficking [31], was highly induced in the ischemic heart at 1 day after MI but gradually suppressed thereafter. These findings indicate that intracellular copper trafficking is impaired along with the loss of myocardial copper in the ischemic heart after prolonged injury.

Furthermore, we found that the expression of secretory copper enzymes, including the ferroxidase family (*CP* and *HEPH*), lysyl oxidase family (*LOX*, *LOXL1*, *LOXL2* and *LOXL3*), and superoxide dismutase 3 (*SOD3*), was reversely increased accompanied by myocardial copper loss, suggesting an increased transfer of intracellular copper to the secretory pathway after prolonged ischemia (Fig. 4D). Taken together, the differential regulation of copper homeostasis-related genes after myocardial ischemic injury may impair copper bioavailability in the ischemic heart, resulting in the selective regulation of HIF-1 signaling after MI.

## Discussion

In this study, we generated a resource database of the myocardial transcriptome in the rhesus monkey heart following ischemic injury across a 4-week time window. We demonstrate that the unique transcriptomic signature of the ischemic heart at 1, 7, and 28 days after LAD ligation fully reflects the pathophysiological manifestation during the progression of MI. Further analysis of dynamic changes in HIF-1 target genes in the ischemic heart identified three subsets of genes with distinct expression patterns specifically involved in processes related to glycolysis, angiogenesis, and ECM remodeling. The temporal fine-tuning of HIF-1 target gene expression after myocardial ischemia reveals a precisely selective regulation of HIF-1 transcriptional activity in the ischemic heart, which is probably attributed to the change in myocardial copper homeostasis.

In the past two decades, global transcriptome analysis by microarray or RNA-seq has been greatly applied not only to explore the molecular mechanism underlying the pathogenesis of ischemic heart diseases but also to identify biomarkers useful for diagnostic, prognostic, and therapeutic purposes. Numerous genes have been recognized as causative or responsive factors in the repair and remodeling of the infarcted heart [32–34]. However, few studies have revealed their temporal changes after MI [35–37]. By analyzing the dynamic changes in the myocardial transcriptome during the progression of MI in the rhesus monkeys, we found a temporal-specific regulation of genes associated with the inflammatory response, cell proliferation, fibrosis and mitochondrial metabolism at different phases of MI. Inflammatory response genes, such as *CXCR1*, a chemokine receptor mainly expressed in neutrophils, were acutely induced after injury and gradually recovered thereafter. This is most likely attributed to the massive infiltration of inflammatory cells in the ischemic heart for the clearance of dead cells and matrix debris after acute injury, and the inflammatory infiltration is programmed to gradually resolve during the subsequent reparative processes [2, 38]. Furthermore, genes associated with cell proliferation were specifically activated 7 days after ischemic injury, accompanied by a marked increase in the cell division marker Ki67. This implies that a large number of cells in the infarcted myocardium, mainly non-myocytes (e.g., fibroblasts and endothelial cells), undergo significant proliferation on day 7 after injury [39]. Notably, increasing evidence suggests that cardiomyocytes also enter the cell cycle after injury and contribute to increased expression of genes involved in cell proliferation, although only a very small number of cells eventually undergo cell division [40]. After long-term myocardial ischemia (28 days after injury), the expression of fibrotic genes (e.g., *COL1A1* and *LOX*) was dramatically enhanced, leading to increased ECM production and deposition, as revealed by immunostaining of type I collagen. In addition, we observed that mitochondrial metabolism-related genes were significantly suppressed 7 days after injury, which may be primarily due to an adaptive metabolic shift in high-energy-demanding cardiomyocytes in response to reduced oxygen and substrate supply in the infarcted myocardium [41, 42]. Another possible explanation is that the proportion of mitochondrial-rich and metabolically active cardiomyocytes in the infarcted area was reduced after 7 days of ischemic injury, resulting in a relative decrease in mitochondrial metabolic gene expression. Interestingly, we also noticed a slightly recovery of the expression of *COX7A1*, a subunit of cytochrome *c* oxidase (C*c*O), in the ischemic myocardium in the IA 28d group compared to the IA 7d group (data not shown). However, the mechanism and biological function of the dynamic changes of *COX7A1* expression during MI progression remains to be further investigated. Taken together, these dynamic transcriptome changes during the progression of MI provide a molecular basis for understanding the pathophysiology of ischemic heart diseases.

Given that tens of thousands of genes involved in various biological processes, such as inflammation, apoptosis, angiogenesis, metabolic reprogramming and fibrosis, are differentially expressed in the ischemic heart [34], the superbly orchestrated regulation of the expression of certain genes at certain phases of MI is critical for the orderly progression of infarct healing. HIF-1 is one of the key transcription regulators in response to myocardial ischemia, regulating the expression of hundreds of genes, most of which are involved in cardiac remodeling [8–10, 43]. However, the specific role of HIF-1 signaling in postinfarction healing remains unclear. We showed here that the expression of HIF-1 target genes following myocardial ischemic injury exhibited dynamic and functional-specific expression patterns. For instance, in the acute phase of MI, HIF-1-mediated glycolytic and angiogenic genes, such as *SLC2A1* and *VEGFA,* were significantly induced in the ischemic heart, while in the prolonged phase of MI, these glycolytic and angiogenic genes were gradually suppressed, but instead, HIF-1-mediated ECM remodeling genes, such as *LOX* and *COL5A1*, were highly activated. This finding indicates that HIF-1 signaling exerts a distinct cardioprotective role in different phases of MI, promotes myocardial adaptation to ischemia by inducing glycolytic and angiogenic genes after acute injury, and prevents cardiac rupture by enhancing the expression of reparative fibrotic genes after prolonged injury. However, what is the main determinant for this dynamic adjustment of HIF-1 signaling during the progression of MI?

To address this question, we measured copper concentrations in the ischemic heart at different phases of MI, since copper selectively regulates the expression of certain HIF-1 target genes by affecting the binding of HIF-1α to their HREs [12, 23]. We found that myocardial copper concentrations did not change significantly after acute injury (within 24 h after MI) but continued to decrease after prolonged injury (7 days after MI). Furthermore, myocardial copper levels exhibited a significantly positive correlation with the expression of HIF-1-mediated glycolytic and angiogenic genes. Previous studies in endothelial cells and cardiomyocytes have shown that copper deprivation by TEPA significantly abolished hypoxia-induced expression of copper-dependent HIF-1 target genes, such as *BNIP3* and *VEGFA* [21–23]. Therefore, the suppression of HIF-1-mediated glycolytic genes and angiogenic genes in the ischemic heart after long-term injury is probably attributed to the loss of myocardial copper. Counterintuitively, a significant negative correlation between myocardial copper levels and the expression of HIF-1-mediated ECM remodeling genes was also observed. A reasonable explanation is that suppression of copper-dependent HIF-1 target genes after prolonged ischemia will in turn enhance the binding of HIF-1α to the HREs of these copper-independent ECM remodeling genes and promote their subsequent transcriptional activation.

Moreover, by analyzing the expression pattern of genes related to copper homeostasis, we found that copper chaperones and copper storage genes were significantly suppressed, while secretory copper enzymes genes were all induced along with the loss of myocardial copper in the ischemic heart after prolonged injury, indicating a redistribution of myocardial copper with the progression of MI. This is supported by previous findings that copper-dependent C*c*O activities were depressed but LOX activities were increased in the heart after long-term ischemic injury [44]. Prevention of myocardial copper loss by cardiac-specific knock out of *Commd1* preserved HIF-1 target *Vegfa* and *Flt1* expression and protected mice hearts from ischemic injury [26]. Thus, perturbation of myocardial copper homeostasis is most likely a key determinant of inhibiting HIF-1-targeted angiogenesis and enhancing ECM remodeling after myocardial infarction. Targeting copper homeostasis for fine-tuning HIF-1 transactivation may represent a novel therapeutic approach for ischemic heart disease. Future research will focus on identifying copper-binding proteins involved in the selective regulation of HIF-1 target gene expression under hypoxia, which could provide more specific therapeutic targets.

In addition to the alteration in myocardial copper homeostasis, differences in the cell-type-specific epigenetic modification of HIF-1 target genes caused by altered myocardial composition in the ischemic heart may also result in the differential expression of HIF-1 target genes. Future studies will combine single-cell sequencing data and spatial transcriptome data after MI to understand the changes and roles of HIF-1 signaling from multiple dimensions.

Considering its cardioprotective role in promoting prosurvival signaling in the heart after acute ischemic injury, HIF-1 has become a promising therapeutic target for ischemic heart disease. Numerous studies have shown that enhancing HIF-1 signaling by overexpressing or stabilizing HIF-1α protein in the heart improves myocardial function and limits the infarct size after acute coronary occlusion [45–51]. However, sustained activation of HIF-1α in the heart eventually results in dilated cardiomyopathy with a variety of histological changes, including myocyte loss and fibrosis [14, 52–54]. Determination of the cutoff point or the threshold at which HIF-1 stops being beneficial and starts its detrimental effects requires a deep deciphering of the molecular underpinnings of HIF-1 signaling during the progression of MI. In the present study, we found that HIF-1-mediated ECM remodeling signaling starts to be activated in the ischemic heart at 7 days after injury. Artificially induced HIF-1α accumulation in the ischemic heart 7 days after MI would specifically enhance the expression of copper-independent ECM remodeling genes, thus resulting in a cardio-deleterious effect, such as cardiac fibrosis. Therefore, the beneficial cutoff point of HIF-1 targeted therapy may be the time prior to the massive loss of myocardial copper after ischemic injury. Alternatively, specifically increasing copper bioavailability in the ischemic heart will probably eliminate the deleterious effect of HIF-1 activation.

On the other hand, we noticed a ~ 50% decrease in copper concentrations in monkey ischemic myocardium on day 7 post-MI, accompanied by marked inhibition of HIF-1 target angiogenic and glycolytic gene expression. Likewise, our previous studies in mice showed that myocardial copper levels were reduced by 50% at 7 days after MI [25], whereas cardiac-specific knockout of *Commd1* restored copper content in ischemic myocardium to 60% of normal levels, resulting in partial restoration of expression of HIF-1 target angiogenic genes such as *Vegfa* and *Flt1* [26]. Therefore, we propose that copper concentrations in the ischemic myocardium may need to be maintained above 60% of normal levels for effective HIF-1-targeted therapy. Since direct measurement of copper concentration in ischemic myocardium for clinical diagnosis and treatment is almost impractical, future studies are required to identify blood biomarkers that are closely correlated to myocardial copper status (i.e., ceruloplasmin [55]), which will provide more feasible reference indicators for HIF-1 targeted therapy.

A major limitation of this study is the lack of protein-level validation and functional evidence on the dynamic expression of HIF-1 target genes during MI progression. Nonetheless, numerous studies over the past few decades dissecting the pathological, molecular, and functional characteristics of ischemic myocardium during MI progression provide conclusive evidence for the findings in this study [8, 16, 56]. However, further functional verification is still needed for the inference using myocardial copper as a reference for HIF-1 targeting therapy.

## Conclusions

This work identifies a dynamic, functional-specific expression pattern of HIF-1 target genes in the ischemic heart during the progression of MI, which is probably attributed to the alteration in myocardial copper homeostasis. Our findings provide transcriptomic insights into the beneficial or deleterious role of HIF-1 in the heart after ischemic injury, which will help determine the beneficial cutoff point of HIF-1 targeted therapy for ischemic heart disease.

## Supplementary Information

**Additional file 1: Fig. S1.** Venn diagram and GO analysis of DEGs at different times after MI. **A** Venn diagram showing the number of overlapping and unique DEGs between different comparisons. **B** GO enrichment analysis of the differential expressed HIF-1 target genes in the ischemic myocardium at 1, 7 and 28 days after MI

**Additional file 2: Fig. S2.** Dynamic expression of HIF-1 target genes in a mouse model of MI. **A** Heatmap visualization of the expression patterns of three distinct sets of HIF-1 target genes, including angiogenesis, glycolysis, and ECM remodeling, during MI progression in mice. RA: remote area; IA: infarcted area. **B** Changes in gene expression of Vegfa, Slc2a1, and Lox were analyzed by RT-qPCR at 1, 7, and 28 days after MI in mice. n=3–4. *, *p* <0.05, significantly different from the corresponding sham-operated controls

**Additional file 3: Table S1.** Differentially expressed genes (DEGs) in the ischemic heart at 1, 7, and 28 days after MI

**Additional file 4: Table S2.** The list of the top 25 DEGs enriched in inflammatory response, cell proliferation, cardiac fibrosis, and mitochondrial metabolism after MI

**Additional file 5: Table S3.** The list of previously validated HIF-1 target genes

**Additional file 6: Table S4.** The list of 89 significantly changed HIF-1 target genes in the ischemic monkey heart

**Additional file 7: Table S5.** The list of HIF-1 target genes positively or negatively correlated with myocardial copper status

## Data Availability

The raw date of RNA-seq is available in NCBI's Sequence Read Archive (SRA) database (accession number: SRP369731).

## Abbreviations

AAS: Atomic absorption spectrophotometer
ADM: Adrenomedullin
ALDOA: Aldolase
ANGPT2: Angiopoietin 2
ATOX1: Antioxidant 1
BNIP3: 19 KDa BCL2/adenovirus E1B protein-interacting protein 3
BSA: Bovine serum albumin
CBP: CREB-binding protein
CCO: Cytochrome c oxidase
CCS: Copper chaperone for superoxide dismutase
CDK1: Cyclin-dependent kinase 1
COL1A1: Collagen type I alpha 1 chain
COL5A1: Collagen type V alpha 1 chain
Commd1: Copper metabolism MURR1 domain1
COQ9: Coenzyme Q9
CP: Ceruloplasmin
CXCR1: C-X-C motif chemokine receptor 1
CXCR4: C-X-C motif chemokine receptor 4
DEGs: Differentially expressed genes
ECM: Extracellular matrix
EDN1: Endothelin 1
ETS: ETS transcription factor family
FLT1: Fms-related tyrosine kinase 1
GAPDH: Glyceraldehyde 3-phosphate dehydrogenase
GO: Gene ontology
GPI: Glucose-6-phosphate isomerase
GSEA: Gene set enrichment analysis
HEPH: Hephaestin
HK1: Hexokinase 1
HK2: Hexokinase 2
HMOX1: Heme oxygenase 1
HIF-1: Hypoxia inducible factor-1
HSP90B1: Heat shock protein 90 beta family member 1
HUVECs: Human umbilical vein endothelial cells
IA: Ischemia-injured area
IGF2: Insulin-like growth factor 2
ITGB2: Integrin beta 2
LAD: Left anterior descending
LDHA: Lactate dehydrogenase A
LOX: Lysyl oxidase
MI: Myocardial infarction
MMP2: Matrix metallopeptidase 2
MMP14: Matrix metallopeptidase 14
P4HA1: Prolyl 4-hydroxylase subunit alpha 1
PCA: Principal component analysis
PDK1: Pyruvate dehydrogenase kinase 1
PGK1: Phosphoglycerate kinase 1
PKM: Pyruvate kinase M1/2
SERPINE1: Plasminogen activator inhibitor-1
SLC2A1: Solute carrier family 2 member 1
SOD3: Superoxide dismutase 3
RNA-seq: RNA-sequencing
TEPA: Tetraethylenepentamine
TGFB3: Transforming growth factor beta 3
TPI1: Triosephosphate isomerase 1
VEGFA: Vascular endothelial growth factor A
VST: Variance stabilizing transformation
VIM: Vimentin
