# Supplementary figures and images for: Dynamic regulation of HIF-1 signaling in the rhesus monkey heart after ischemic injury
Source: BMC Cardiovasc Disord. 2022 Sep 11;22:407. doi: 10.1186/s12872-022-02841-0 (PMC9464399; doi:10.1186/s12872-022-02841-0)

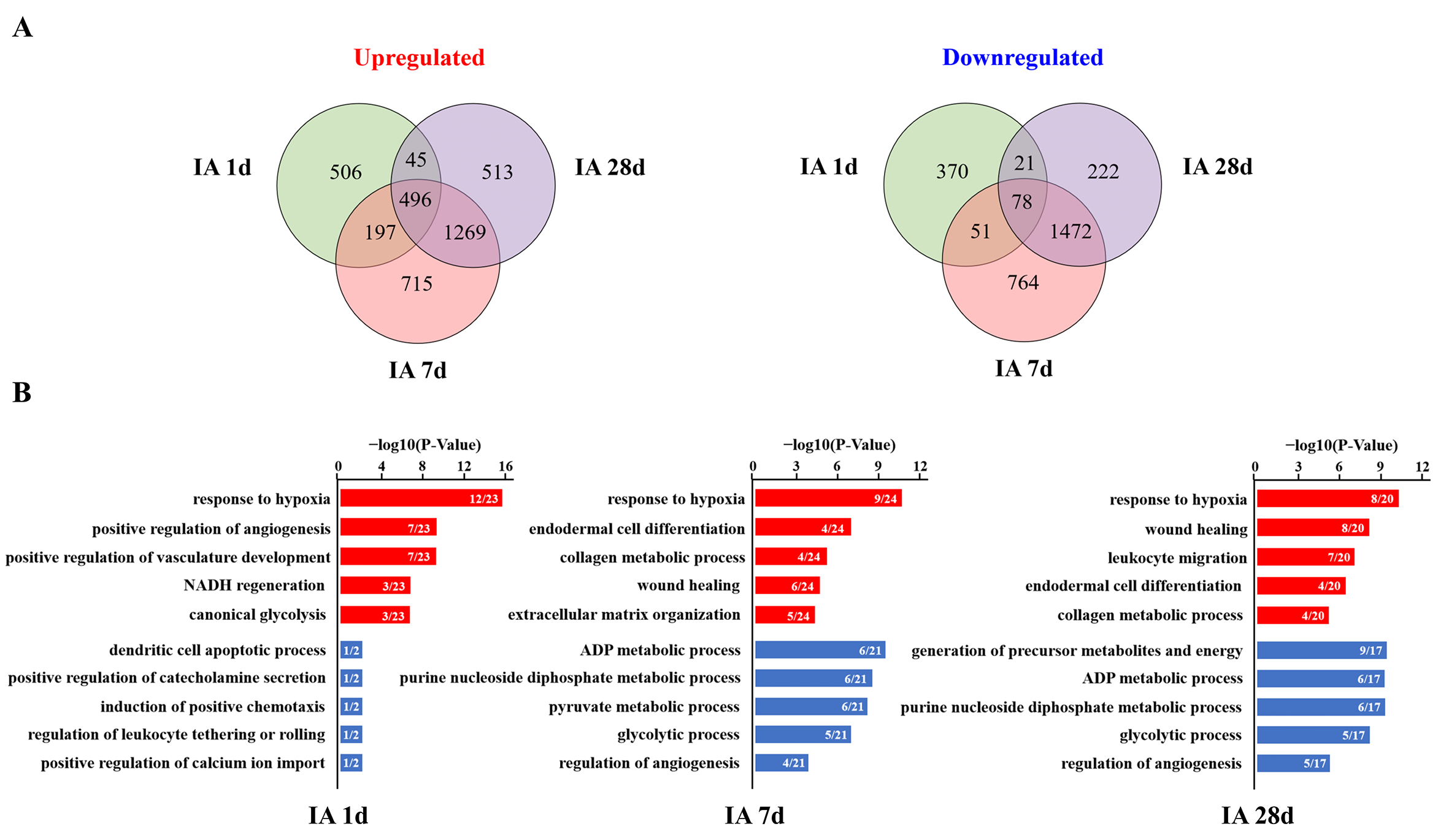

Supplement: Supplementary file 1 — Additional file 1: Fig. S1. Venn diagram and GO analysis of DEGs at different times after MI. A Venn diagram showing the number of overlapping and unique DEGs between different comparisons. B GO enrichment analysis of the differential expressed HIF-1 target genes in the ischemic myocardium at 1, 7 and 28 days after MI [file 12872_2022_2841_MOESM1_ESM.tif]

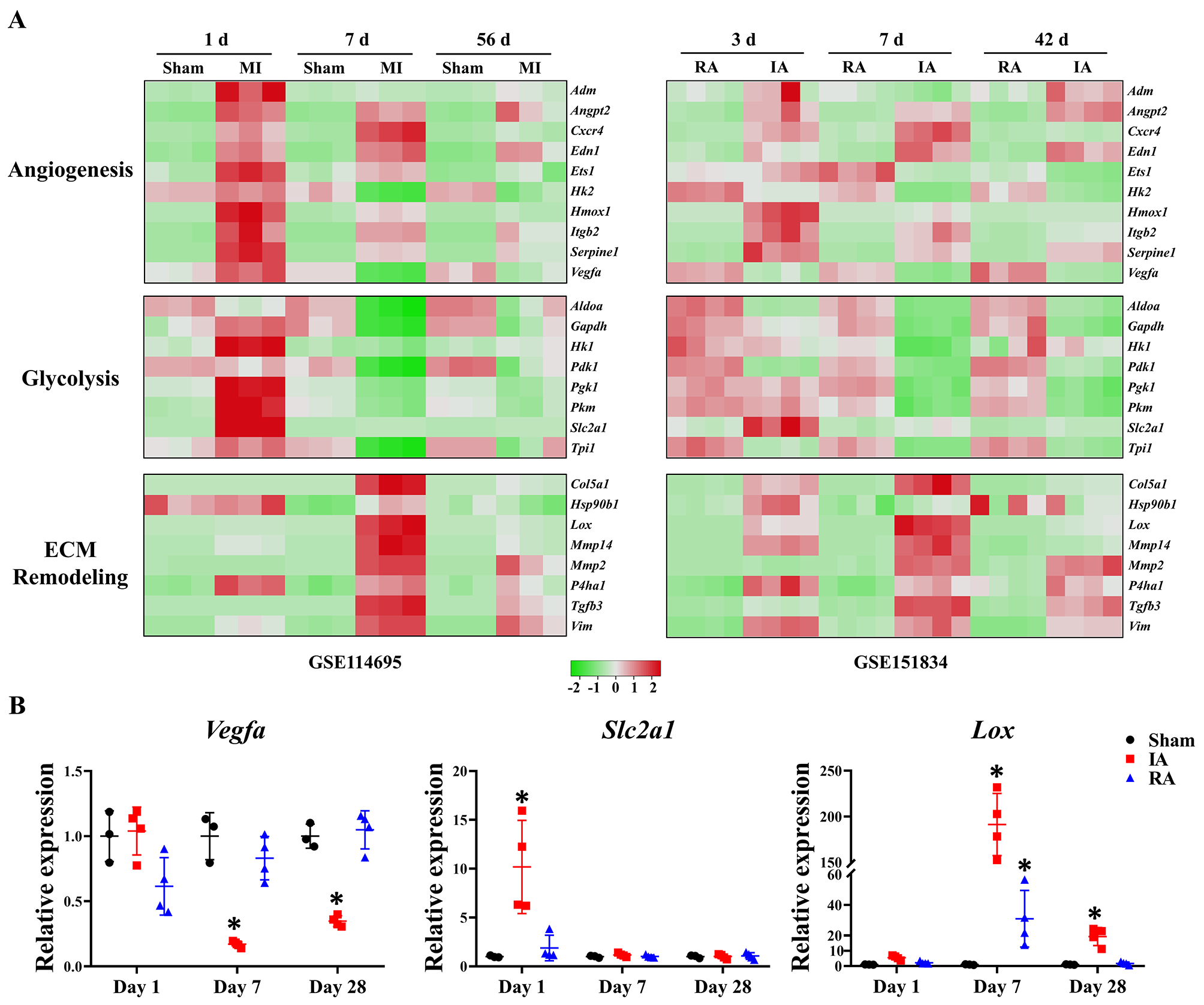

Supplement: Supplementary file 2 — Additional file 2: Fig. S2. Dynamic expression of HIF-1 target genes in a mouse model of MI. A Heatmap visualization of the expression patterns of three distinct sets of HIF-1 target genes, including angiogenesis, glycolysis, and ECM remodeling, during MI progression in mice. RA: remote area; IA: infarcted area. B Changes in gene expression of Vegfa, Slc2a1, and Lox were analyzed by RT-qPCR at 1, 7, and 28 days after MI in mice. n=3–4. *, p <0.05, significantly different from the corresponding sham-operated controls [file 12872_2022_2841_MOESM2_ESM.tif]
